# Combining Amplitude Spectrum Area with Previous Shock Information Using Neural Networks Improves Prediction Performance of Defibrillation Outcome for Subsequent Shocks in Out-Of-Hospital Cardiac Arrest Patients

**DOI:** 10.1371/journal.pone.0149115

**Published:** 2016-02-10

**Authors:** Mi He, Yubao Lu, Lei Zhang, Hehua Zhang, Yushun Gong, Yongqin Li

**Affiliations:** 1 School of Biomedical Engineering, Third Military Medical University, Chongqing 400038, China; 2 Emergency Department, Xinqiao Hospital, Third Military Medical University, Chongqing 400038, China; 3 Emergency Department, Southwest Hospital, Third Military Medical University, Chongqing 400038, China; 4 Department of Medical Engineering, Daping Hospital & Research Institute of Surgery, Third Military Medical University, Chongqing 400042, China; Temple University, UNITED STATES

## Abstract

**Objective:**

Quantitative ventricular fibrillation (VF) waveform analysis is a potentially powerful tool to optimize defibrillation. However, whether combining VF features with additional attributes that related to the previous shock could enhance the prediction performance for subsequent shocks is still uncertain.

**Methods:**

A total of 528 defibrillation shocks from 199 patients experienced out-of-hospital cardiac arrest were analyzed in this study. VF waveform was quantified using amplitude spectrum area (AMSA) from defibrillator's ECG recordings prior to each shock. Combinations of AMSA with previous shock index (PSI) or/and change of AMSA (ΔAMSA) between successive shocks were exercised through a training dataset including 255shocks from 99patientswith neural networks. Performance of the combination methods were compared with AMSA based single feature prediction by area under receiver operating characteristic curve(AUC), sensitivity, positive predictive value (PPV), negative predictive value (NPV) and prediction accuracy (PA) through a validation dataset that was consisted of 273 shocks from 100patients.

**Results:**

A total of61 (61.0%) patients required subsequent shocks (N = 173) in the validation dataset. Combining AMSA with PSI and ΔAMSA obtained highest AUC (0.904 vs. 0.819, *p*<0.001) among different combination approaches for subsequent shocks. Sensitivity (76.5% vs. 35.3%, *p*<0.001), NPV (90.2% vs. 76.9%, *p* = 0.007) and PA (86.1% vs. 74.0%, *p* = 0.005)were greatly improved compared with AMSA based single feature prediction with a threshold of 90% specificity.

**Conclusion:**

In this retrospective study, combining AMSA with previous shock information using neural networks greatly improves prediction performance of defibrillation outcome for subsequent shocks.

## Introduction

Cardiac arrest is a major public health problem around the world and ventricular fibrillation (VF) is a common presenting heart rhythm during cardiac arrest [1]. Early cardiopulmonary resuscitation (CPR) together with early defibrillation is a key point in the chainof survival for cardiac arrest patients with VF [2,3]. However, there is no consensus on the balance between CPR and defibrillations, particularly for out-of-hospital cardiac arrest (OHCA) victims with a long response time since not all patients in VF might benefit from being treated in the same manner of the time-based protocols [4–6]. Optimizing timing of defibrillation can reduce the unnecessary interruptions of chest compression and decrease the severity of post-resuscitation myocardial dysfunction by reducing the numbers of failed or unnecessary shocks, which, therefore, has the potential to improve overall survival from cardiac arrest [7].

Quantitative electrocardiogram (ECG) waveform analysis provides a non-invasive reflection of the state of the myocardium during resuscitation and is a potentially powerful tool to optimize CPR intervention, i.e., chest compression or defibrillation[8]. During the last two decades, numerous features have been developed from the ECG of a shockable rhythm prior to defibrillation and used to predict the outcome of defibrillation[9–13]. However, a sensitivity of around 33–45% with a specificity of 90% for these features indicated that single measure extracted from VF waveform might have reached its maximum prediction power [14]. New strategies are still required to develop more accurate methods for shock outcome prediction.

As combining multiple features may improve the predictive accuracy by offering complementary information, several studies have been attempted to combine different features to enhance the predictive performance using the machine learning theory[15–19]. Earlier trials combing VF features with clinical arrest factors such as patients’ age, sex and ambulance response time, showed no significant improvement of prediction performance[15]. Combing within-patient correlation with VF feature presented a trend towards predicting improvement[16]. Studies about whether combing several single VF features could improve prediction capability for defibrillation did not reach consistent results[17–19]. Combing VF with end-tidal carbon dioxide (PetCO2) could significantly improve the performance[20],butPetCO2 measurements are not widely available, especially in the scenario of OHCA.

The purpose of the present study was to investigate whether combing VF waveform feature amplitude spectrum area (AMSA)with previous shock information using neural networks could improve the performance of defibrillation prediction in OHCA patients. AMSA was used as the quantitative VF measure because it has been demonstrated to be one of the most accurate predictors for defibrillation outcome in both animal and retrospective clinical studies[13,14].

## Material and Methods

### Data collection

Data were collected from emergency departments of Southwest Hospital and Xinqiao Hospital in Chongqing between Jan 2012 and Feb 2014. Ethical approvals were obtained from the ethics committee of the First Affiliated Hospital (Southwest Hospital) of Third Military Medical University and the ethical Committee of Second Affiliated Hospital (Xinqiao Hospital) of Third Military Medical University. The informed consent was waived by the committees because this was a retrospective and observational study and the data were automatically collected by the defibrillators at the time the pads were placed on the chest. ECG were recorded for victims experienced OHCA and CPR by defibrillator through two adhesive adult defibrillation/pacing pads. The electronic data did not contain any patient’s identifiable information. A total of 528 shocks from 199 non-traumatic adult patients with VF as the recorded initial rhythm and with determinable pre-shock and post-shock rhythms were enrolled. Among the 199 patients, 131 were treated with Philips defibrillators (Philips MRx, Philips Medical Systems, Seattle, WA, USA) and 68 patients were treated with ZOLL defibrillators (M-Series, ZOLL Medical Corporation, Chelmsford, MA, USA).

The post-shock rhythms were categorized as VF (a disorganized rhythm, with a median amplitude of >100 uV), asystole (<100 uV) or organized rhythm by two medical doctors. Defibrillation outcome was regarded as successful or return of a potentially perfusing rhythm if an organized rhythm was present within 60 seconds after the shock, and had a rate of 40 beats/min or greater.^21^Shocks were divided into first and subsequent ones[21].

### Data analysis

The analysis was performed offline through user-designed software based on Matlab (The MathWorks, Inc., Natick, MA, USA). Data were randomized to a training set that including 255shocks from 99 patients and a validation set that including273shocks from 100 patients. An episode of 2.05 seconds ending at 0.5 second prior to each defibrillation attempt was selected for analysis. In pre-processing, ECGs were filtered by a band-pass filter (2–48Hz) to remove baseline drifting and high frequency interference. The filtered ECG signals were converted from time to frequency domain by fast Fourier transformation. AMSA was calculated as the sum of the products of individual frequencies and their amplitudes, i.e. *AMSA* = ∑*A*_*i*_ × *F*_*i*_ where *A*_*i*_ represented the amplitude at ^*i*^th frequency *F*_*i*_.

For each patient, previous shock index (PSI)and change of AMSA(ΔAMSA) were obtained for each shock during the course of resuscitation. PSI was defined as binary variable based on outcome of previous shock:

$$PSI_{k}=\{\begin{array}{c} 1,\text{ if }k>1\text{ and previous shock was successful}\\ -1,\text{ if }k>1\text{ and previous shock was unsuccessful}\\ 0,\text{ if }k=1 \end{array}$$ (1)

ΔAMSA was defined as the difference of AMSA values between consecutive shocks:

$$\Delta AMSA_{k}=\{\begin{array}{c} AMSA_{k}-AMSA_{k-1},\text{ if }k>1\\ 0,\text{ if }k=1 \end{array}$$ (2)

**Fig 1:**
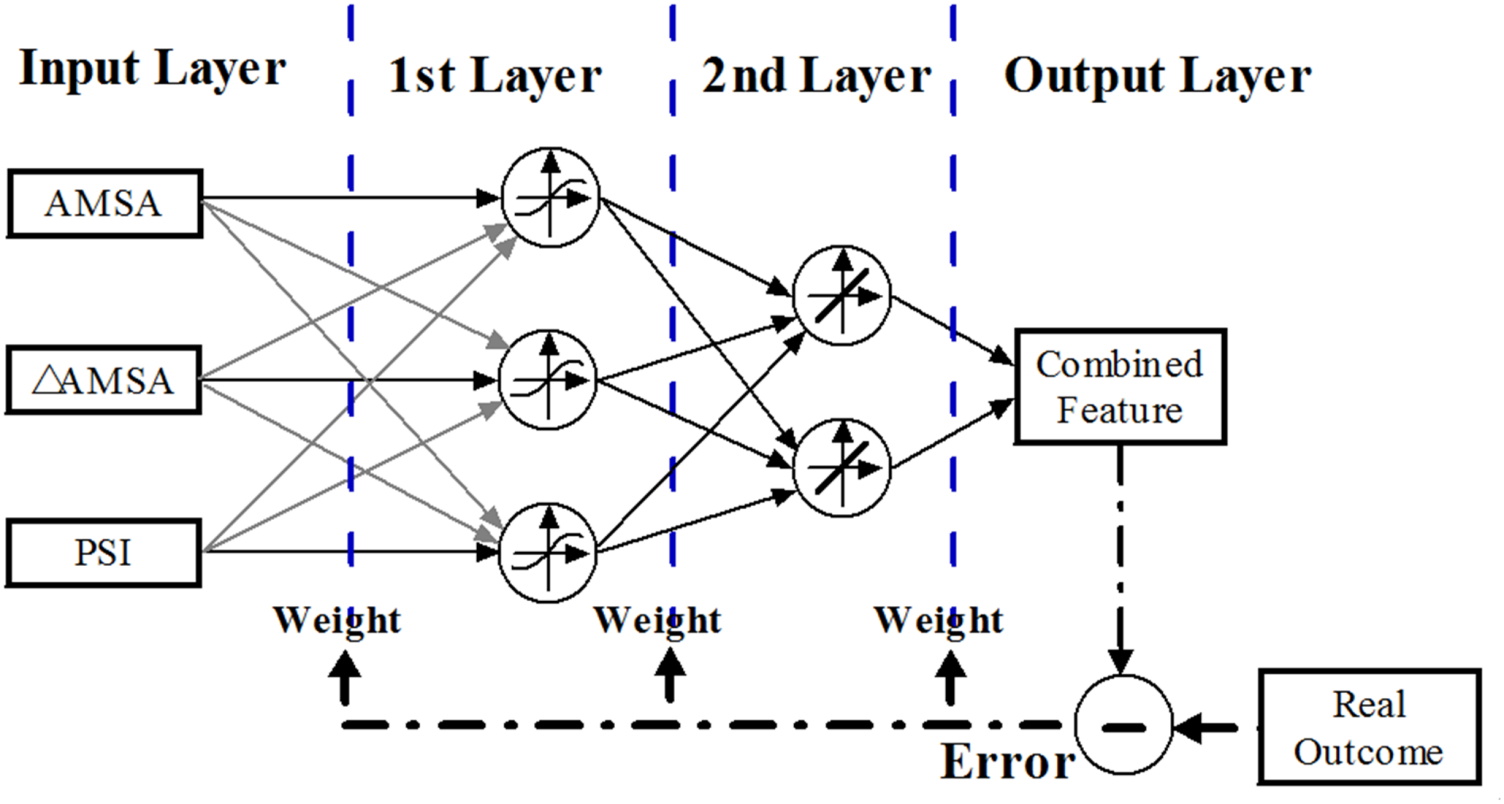
The schematic layout of the BP neural network structure and its training process.

Three different combination approaches: AMSA and PSI (C1), AMSA and ΔAMSA (C2),AMSA together with PSI andΔAMSA (C3) were tested and compared with AMSA based single feature perdition. A back propagation(BP) neural network with a feed forward structure was employed for the combination[18]. The schematic layout of the neural network structure and its training process was shown in Fig 1. The BP neural network adopted the Bayesian regularization training function, two hidden layers with three sigmoid and two linear transfer functions respectively. These parameters were optimized to avoid underfitting or overfitting by evaluating the predicting performance of the neural network. The training set was used to optimize the weights of the network and the validation set was used to assess predictive power. To examine whether the proposed strategy was independent of machine learning method, the logistic regression model was also used to evaluate the predictability as each extra predictor variable was added to AMSA.

Receive operator characteristic (ROC) curve was constructed and a threshold when specificity equaled to 90% for training data was used to result in a binary decision. The prediction power was assessed by area under ROC curve (AUC), sensitivity, positive predictive value (PPV), negative predictive value (NPV) and prediction accuracy (PA).

### Statistical analysis

Continuous variables were expressed as mean±standard deviation. Differences in AMSA between successful and unsuccessful shocks were compared by independent-samples *t*-test. For subsequent shocks, Hausman's test was used to determine the correct specification of the model, fixed versus random effects[22]; single variable logistic regression was used to calculate odds ratio (OR) and 95% confidential interval (CI) between prediction features (AMSA, PSI andΔAMSA, respectively)and outcome with the appropriate model; nonparametric correlation coefficient(Kendall's tau) was used to evaluate the association between these variables. AUCs were compared using Z-test. Chi-square test was employed to distinguish differences among sensitivity, NPV, PPV and PA of the different predicting methods. A final *p* value < 0.05 was considered statistically significant.

## Results

Among 199 patients who received an initial shock, 128 (64.3%) required subsequent shocks (N = 329). The defibrillation success rate was 37.7% and AMSA was significantly higher prior to successful first shocks (15.9±9.0 vs. 9.3±5.8 mVHz, *p*<0.001). For subsequent shocks, 94 were successful (28.6%)while 235 were failed (71.4%). AMSA (16.8±8.7 vs. 8.3±5.6 mVHz, *p*<0.001), PSI (0.26±0.97 vs. -0.81±0.58, *p*<0.001) and ΔAMSA (1.2±6.1 vs. -0.3±3.7 mVHz, *p* = 0.029) were all significantly higher for successful subsequent shocks compared with unsuccessful ones. Hausman's test revealed a random effect model was better than its fixed counterpart(*p*<0.0001). Single variable logistic regression with a random effect model indicated that AMSA (OR = 1.030, 95% CI: 1.024–1.036, *p*<0.001), PSI (OR = 1.340, 95% CI: 1.278–1.404, *p*<0.001)and ΔAMSA (OR = 1.015, 95% CI: 1.004–1.026, *p* = 0.007) were independent predictor of defibrillation success for subsequent shocks. The correlation coefficients were 0.377 (*p*<0.001) between AMSA and PSI, 0.214 (*p*<0.001) between AMSA and ΔAMSA, -0.003 between ΔAMSA and PSI (*p* = 0.940).

**Fig 2:**
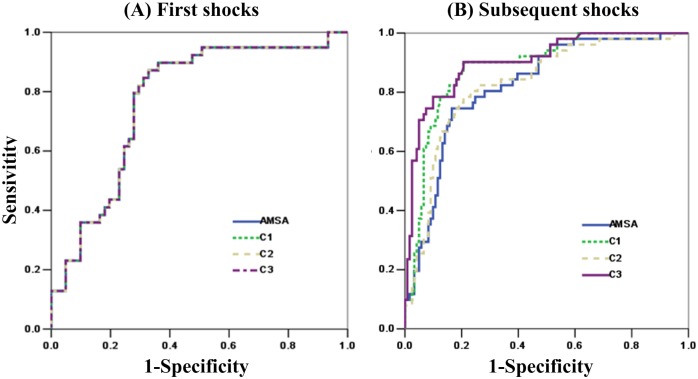
Receive operator characteristic curves for defibrillation outcome prediction of first (A) (N = 100) and subsequent (B) (N = 173) shocks in validation dataset.

A total of 67 (67.7%) patients in training set and 61 (61.0%) patients in validation set received subsequent shocks. Fig 2 shows the ROC curves of shock outcome prediction with the three different combination approaches and AMSA in the validation set. For first shocks, AUC was unchanged (0.770) when different combination approaches were applied. For subsequent shocks, AUC was significantly increased when combinations C1 (0.883 vs. 0.819, *p* = 0.005) and C3 (0.904 vs. 0.819, *p*<0.001) were employed, but no statistical difference was observed between C2 and AMSA (0.825 vs. 0.819, *p* = 0.818).

The prediction performances of different combination approaches and AMSA for subsequent shocks in validation set by the BP neural network method were listed in Table 1. Compared with AMSA based single feature prediction, C1 and C3 remarkably improved the sensitivity (C1: 68.6% vs. 35.5%, *p*<0.001; C3: 76.5% vs. 35.3%, *p*<0.001), NPV (C1: 87.4% vs. 76.9%, *p* = 0.026; C3: 90.2% vs. 76.9%, *p* = 0.007) and PA (C1: 84.4% vs. 74.0%, *p* = 0.017; C3: 86.1% vs. 74.0%, *p* = 0.005)with a threshold of 90% specificity. However, no statistical differences were observed in prediction power whenC2 was employed.

**Table 1:** Performance of amplitude spectrum area (AMSA) and three different combination strategies for the prediction of subsequent shocks in validation data (51 successful and 122 failed shocks) by the BP neural network.

	AUC	Sensitivity(%)	Specificity(%)	NPV(%)	PPV(%)	PA(%)
**AMSA**	0.819	35.3	90.2	76.9	60.0	74.0
**C1**	0.883**	68.6##	91.0	87.4#	76.1	84.4#
**C2**	0.825	49.0	90.2	80.9	67.6	78.0
**C3**	0.904**	76.5##	90.2	90.2##	76.5	86.1##

C1: AMSA+PSI;C2: AMSA+ΔAMSA; C3: AMSA+PSI+ΔAMSA;PSI: previous shock index; AUC: area under receiver operating characteristic curve; NPV: negative predictive value; PPV: positive predictive value; PA: prediction accuracy.

**p<0.01 compared with AMSA using Z-test;

^#, ##^*p*<0.05 and *p*<0.01 compared with AMSA using Chi-squared test.

The prediction performances of different combination approaches and AMSA for subsequent shocks in validation set by the logistic regression method with a random effect model were listed in Table 2. Similar with the application of BP network, AUC, sensitivity, NPV and PA were also significantly (*p*<0.05) improved when the logistic regression model was used.

**Table 2:** Performance of amplitude spectrum area (AMSA) and three different combination strategies for the prediction of subsequent shocks in validation data (51 successful and 122 failed shocks) using the logistic regression method with a random effect model.

	AUC	Sensitivity(%)	Specificity(%)	NPV(%)	PPV(%)	PA(%)
**AMSA**	0.821	74.1	90.2	90.2	77.1	60.0
**C1**	0.891**	70.6##	91.1	87.4#	88.2	85.1#
**C2**	0.825	49.0	90.2	80.9	81.0	78.2
**C3**	0.892**	72.5##	91.1	90.2#	88.9	85.6##

C1: AMSA+PSI;C2: AMSA+ΔAMSA; C3: AMSA+PSI+ΔAMSA;PSI: previous shock index; AUC: area under receiver operating characteristic curve; NPV: negative predictive value; PPV: positive predictive value; PA: prediction accuracy.

**p<0.01 compared with AMSA using Z-test;

^#, ##^*p*<0.05 and *p*<0.01 compared with AMSA using Chi-squared test.

## Discussion

In this retrospective study, we demonstrated that combining quantitative VF waveform feature AMSA with previous shock outcome and change of AMSA between consecutive shocks using neural networks significantly improved the performance of defibrillation prediction for subsequent shocks.

Features of the VF waveform, such as frequency and amplitude related AMSA, correlated with coronary perfusion pressure and has been described as a method to predict when a VF is likely to be shocked into a pulse generating rhythm, thereby reducing pauses in chest compressions and potential myocardial damage by limiting redundant shocks[8,14,21,23]. However, factors that influencing VF characteristics might affect the probability of defibrillation success and therefore the performance of predictability of these features. In animal studies, Indik et al.[24,25]observed that VF waveform characteristics were altered substantially in animals with myocardial infarction and heart failure compared to structurally normal controls. Olasveengen et al.[26] confirmed that acute myocardial infarction (AMI) patients have depressed AMSA compared to patients without AMI in 101 OHCA victims. Strohmenger et al.[27] found that vasopressin increased median frequency of VF more than epinephrine and improved the chances of successful defibrillation in a porcine model of cardiac arrest. Sherman et al.[28] reported that adrenergic blockade prior to VF induction affected quantitative measures of VF waveform and might limit the ability of such measures to predict downtime or defibrillation outcome in animal study. Shanmugasundaram et al.[29] investigated the effects of VF type on the performance of AMSA and slope for the prediction of defibrillation success in 44 OHCA patients with VF as initial rhythm and reported that AMSA and slope predicted defibrillation in shock-resistant VF but not in recurrent VF.

In order to identify approaches to improve the accuracy of shock outcome prediction, Gundersen et al.[15]analyzed shock outcome prediction data with a mixed effects logistic regression model but did not find significant improvement by including the peri-arrest factors age, sex, presenting rhythm, presence of bystander CPR and ambulance response time with VF features in the model. Gundersen et al.[16] then developed an algorithm to re-estimate the patient-dependent relationship between predictive feature value and probability of ROSC by incorporating the information from prior shocks using the same model. Although the results indicated that it was possible to improve current shock prediction methods by using an updating algorithm capable of learning from previous shocks, the improvements were relatively modest and therefore whether using within-patient information could improve the accuracy of shock prediction was still need to be investigated.

Neural networks, which have been used to predict defibrillation success using ECG signal or multiple VF features[18,30],have not been evaluated for combining VF feature with patient related previous shock information. In the current study, quantitative VF waveform feature AMSA was combined with PSI, ΔAMSA, PSI and ΔAMSA using neural networks for the prediction of defibrillation outcome. PSI was used because previous clinical studies demonstrated that there was within-patient correlation between the predictive feature value and probability of defibrillation success and repeated shocks in one patient were considered as dependent events[16,29]. Consistent with these studies, our results showed that PSI wasan independent predictor of defibrillation success for subsequent shocks. Independent testing in the validation set revealed that AUC, sensitivity, NPV and PA were improved by 7.8%, 94.3%, 13.7% and 14.1% when AMSA was combined with PSI for the prediction of subsequent shocks. Since difference in AMSA value between consecutive shocks was associated with depth of chest compression and favorable neurologic survival[21,31],we evaluated the performance of combining AMSA and ΔAMSA using neural networks. Although AUC, sensitivity, NPV and PA values were increased compared with AMSA based single feature prediction, the improvement was insignificant. When AMSA was combined together with PSI and ΔAMSA, the performance was further improved for subsequent shocks. The improvement was 10.4%, 116.7%, 17.3% and 16.4% for AUC, sensitivity, NPV and PA respectively. The improvements obtained by combining AMSA with previous shock information using neural networks could be explained by the complementary information provided by the selected parameters. AMSA, which describes the amplitude-weighted mean frequency of VF waveform, reflects with heart's metabolic state[8,10,21,23]. PSI, which denotes the outcome of the previous defibrillation, is associated with the within-patient information such as type of VF.[16,25][26,29],ΔAMSA, which indicates how VF waveform changes over the course of resuscitation, is correlated with quality of CPR[21]. The lower correlation coefficients between each variables confirmed that PSI and ΔAMSA provided additional information in addition to AMSA. As neural networks offered a powerful framework to adjust the weight of input variants and to merge several single features to a combined one[18,30],the prediction performance of defibrillation outcome for subsequent shocks was therefore significantly improved by combining AMSA with PSI and ΔAMSA using neural networks. The comparable results obtained by applying the logistic regression model indicated that the proposed strategy was independent of machine learning method.

To implement the current strategy in a clinical setting, previous shock information, including outcome of previous shock and change of VF features need to be updated real-time over the course of resuscitation. The digital signal processing capability owned by current generation of automated external defibrillators (AEDs), such as real-time ECG acquisition, rhythm analysis and CPR quality evaluation, enables previous shock information to be saved and the predictive classifier to be updated automatically[32]. Another concern for the implementation of the algorithm is that artifacts produced by chest compression may preclude reliable rhythm analysis and accurate VF waveform analysis during CPR, and therefore degrade the prediction accuracy[33]. However, recent advances in ECG signal processing techniques have made it possible to perform accurate rhythm classification and waveform analysis without interrupting CPR[34–37]. Considering that more than half OHCA patients require repeated defibrillations during resuscitation effort[38,39], the proposed method therefore provides a potential to further enhance resuscitative strategies and improve patient outcome.

This study has a number of limitations. First, in this retrospective study, we did not have phenotypic data of the patient, so the individual difference between successful and unsuccessful resuscitation patients was unknown. Second, the current strategy is only demonstrated to improve the predictability of defibrillation success for subsequent shocks, we anticipate that combining VF feature with other physiological signals, such as PetCO2 and oxygen saturation can improve the performance for first shocks. Third, the proposed method was only shown to be helpful in predicting successful defibrillation as defined in the current study, but whether it is associated long term survival has not been investigated. Forth, this is a retrospective analysis of a small cohort of OHCA patient with an initial rhythm of VF, and further work will be required to validate the clinical usefulness of the current method.

## Conclusion

In this retrospective study, combining AMSA with previous shock information using neural networks greatly improves prediction performance of defibrillation outcome for subsequent shocks. The proposed method, therefore, may provide a real-time strategy for individual resuscitation especially in subsequent attempts to achieve optimal defibrillation.

## Supporting Information

S1 Data AMSA, PSI, ΔAMSA and shock outcome of training dataset and the neural network outcome of validation dataset after combing AMSA with PSI and ΔAMSA. (XLSX)
